# Investigating Associated Factors of Emotional Intelligence (EI) and Its Relationship with Health-Promoting Lifestyles Among Prelicensure Nursing Students

**DOI:** 10.3390/nursrep16020070

**Published:** 2026-02-16

**Authors:** Joanna Hiu Ki Ko, Daniel Yee Tak Fong

**Affiliations:** School of Nursing, The University of Hong Kong, Hong Kong, China; dytfong@hku.hk

**Keywords:** nursing students, nursing education, interpersonal relations, spiritual growth, sleeping quality

## Abstract

**Background/objectives:** Emotional intelligence (EI) plays an important role in nursing education by supporting competencies such as communication, leadership, resilience, and clinical performance. In contemporary nursing education, students face increasing academic, clinical, and emotional demands, highlighting the need to identify modifiable factors that may be associated with EI and can inform student support strategies. Despite extensive EI research, evidence remains limited and inconsistent regarding how specific health-promoting lifestyle domains and sleep quality relate to EI among prelicensure nursing students. This study aimed to examine factors associated with EI and its relationship with health behaviors among prelicensure nursing students. **Methods:** A cross-sectional quantitative design was used. A convenience sample of 287 prelicensure nursing students from a local nursing school completed self-report questionnaires: the Schutte Self-report Emotional Intelligence Scale (SSEIS), the Health-Promoting Lifestyle Profile II (HPLP-II), and the Pittsburgh Sleep Quality Index (PSQI). **Results:** In structured multiphase regression, HPLP-II interpersonal relations (B = 4.42, 95% CI = 1.44 to 7.50, *p* = 0.004) and spiritual growth (B = 6.59, 95% CI = 3.81 to 9.37, *p* < 0.001) were positively associated with EI. Poor sleep quality (PSQI > 5) was negatively associated with EI (B = −1.95, 95% CI = −3.88 to −0.01, *p* = 0.049). **Conclusions:** Interpersonal relations, spiritual growth, and sleep quality were associated with EI among prelicensure nursing students. These factors may be relevant to consider when designing student support and EI-related educational initiatives; however, longitudinal and intervention studies are needed to clarify directionality and causality.

## 1. Introduction

Emotional intelligence (EI), as defined by Salovey and Mayer in 1990 [1], encompasses the capacity to monitor one’s own emotions as well as those of others, to interpret emotional cues effectively, and to use this information to guide cognitive processes and behavioral responses. Evidence suggests that EI plays a significant role in enhancing academic learning, clinical competence, and even patient outcomes [2,3,4]. Beyond influencing individual behavior, EI has also been identified as a determinant of organizational success [5]. In nursing, the ability to regulate and manage emotions is positively associated with critical professional attributes, including critical thinking, leadership, resilience, and empathy [6,7,8,9,10,11,12]. Conversely, lower levels of EI are linked with increased perceived stress among students, potentially impacting both academic and clinical performance [13].

Given the significant role of EI in both the personal and professional development of prelicensure nursing students, identifying the factors that influence EI is essential for designing effective interventions to enhance it. Among these, social support has consistently emerged as a positive contributor. Students who receive adequate support from family and peers tend to demonstrate higher levels of EI, which in turn supports their academic and clinical performance [14]. Clinical exposure is another frequently examined variable, though findings remain inconsistent. Some research suggests that clinical experience does not significantly alter EI levels, even when training programs are implemented [15]. Other studies have found that while healthcare experience may not reach statistical significance, it can contribute to the variance in EI outcomes [16].

Although EI has been widely studied in nursing education, contemporary training environments continue to evolve. Nursing students frequently manage emotionally demanding clinical experiences alongside intensive academic workloads and complex interpersonal interactions with peers, educators, patients, and families. In many settings, healthcare and education are also increasingly influenced by digitalization and rapid system change, which may further amplify the emotional labor required in training and early professional development. Within this context, it remains practically important to identify modifiable factors that may co-occur with EI and can be targeted through student support and educational design.

Socio-demographic variables such as gender and age have yielded mixed findings. For instance, some studies report no significant correlation between gender and EI, whereas others suggest that male nursing students may exhibit higher EI levels than their female counterparts [16]. Similarly, while several studies indicate a positive association between age and EI, others argue that age may only enhance certain EI components, such as emotional perception and reasoning, without improving emotional regulation or expression [17].

There has been a discussion on the potential association of health-promoting lifestyles with EI among prelicensure nursing students [18,19,20], but the evidence remained limited. Some nutritional substances, including folate and dietary oil, are believed to help to enhance EI [21]. Eating disorders may even escalate poor expression and emotional regulation. However, there was only a study of 63 undergraduate nursing students in the United States showing EI was associated with nutrition [19]. Further, physical exercise is believed to be promoting positive and pleasant moods as well as elevating the sense of happiness [18]. Such a proposition was observed with evidence in Taiwan [22] among university students in general. In nursing education, EI was not found significantly associated with physical exercise [19]. However, participants in other faculties had opined the exercise preference of running, doing yoga, and dancing as a stress relief method for better control of emotions. In addition, since sleep deprivation would cause metabolic changes in the cerebrum, leading to delayed responses of emotions and affective dysregulation due to mild prefrontal lobe malfunction, good sleep hygiene is also believed to be closely linked with emotions [23]. However, there has been limited research to examine the relationships between EI and sleep patterns among nursing students. Most of the literature has focused on general university students and showed poor sleep quality led to low conscientiousness and low EI [24,25]. While various other factors have been explored in the literature, their influence on EI remains inconclusive. These discrepancies highlight the need for further empirical investigation to clarify the relative impact of these factors on EI development in nursing education. Therefore, this study aimed to investigate the associated factors towards EI, as well as its relationship with health behaviors among nursing students, to determine the components of EI enhancing programs for them in the coming future.

There has been discussion on the potential association between EI and health-promoting lifestyles among prelicensure nursing students, yet the evidence remains limited and findings are mixed across health domains. Existing studies have examined selected behaviors such as nutrition and physical activity with inconsistent results, and relatively few studies have examined sleep quality in relation to EI specifically among nursing students. Given that sleep disturbance is common in student populations and may relate to emotional functioning, further empirical work is warranted to clarify whether sleep quality and specific health-promoting lifestyle domains (e.g., interpersonal relations and spiritual growth) are associated with EI in this group. These gaps highlight the need for additional empirical evidence to inform educational and student-support approaches in nursing programs.

Therefore, this study aimed to investigate factors associated with EI and examine its relationship with health-promoting lifestyles and sleep patterns among prelicensure nursing students, to inform the future development of EI-supportive educational strategies.

## 2. Methods

### 2.1. Participants

A local nursing school was selected for subject recruitment. Students who were (1) aged 18 or above; and (2) studied in both full-time and part-time programs were included. The convenience sampling method was used for subject recruitment after explaining the aim of the study. An information sheet with study details was given and informed consent was signed by subjects after they fully understood the potential benefits and risks of the study.

The sample size calculation was based on the data obtained from a local study. Standard deviation (SD) taken as reference was equal to 12 [26]. According to Lenth’s power calculator [27], at least 140 students would be required under the condition of error margin was 2 in a 95% confidence interval. There were approximately 15 factors to be measured in the study. According to Rule of Thumb, the sample size should at least be 150 [28]. Taking possibly incomplete questionnaires into account, the number of samples required is set as 200.

### 2.2. Ethical Considerations

The study was approved by Institutional Review Board of The University of Hong Kong (Approval no. UW 18-518) on 27 September 2018 and Research Ethics Committee of Hong Kong Sanatorium & Hospital (Approval no. REC-2018-15) 27 November 2018 respectively. The participants were given an information sheet to understand the purpose and benefits as well as potential risks of the study. Then, they were asked to sign a written consent before filling in the questionnaire. The data collection procedure was anonymous.

### 2.3. Data Collection Procedures

This was a cross-sectional study among pre-licensure nursing students using structured and self-completed questionnaires for assessing EI, health-promoting lifestyles, and sleep patterns. There was a total of 5 nurse educators responsible for the data collection procedures. Subjects were given a set of questionnaires together with an envelope. They were asked to complete the questionnaires, which approximately took 20 min, and seal the envelope after putting the questionnaires in it. The sealed envelopes were directly submitted to the nurse educators for protecting confidentiality. The whole data collection period lasted from 1 December 2018 to 28 February 2019.

### 2.4. Measurements

Participants completed a set of self-reported questionnaires consisting of four parts:

#### 2.4.1. The Schutte Self-Report Emotional Intelligence Scale (SSEIS)

The SSEIS is a well-known questionnaire assessing EI level with 33 items. It was developed based on Mayer and Salovey’s (1997) model of EI [29]. The questions use a 5-point Likert scale for rating, with 1 indicating strongly disagree and 5 indicating strongly agree. The score range was from 33 to 165. A higher total score obtained from participants indicated a higher level of EI. The test–retest reliability and Cronbach’s α were 0.78 [30] and 0.88, respectively [31].

#### 2.4.2. Health-Promoting Lifestyle Profile II (HPLP-II)

HPLP-II is an instrument measuring different health-promoting behaviors by using 52 questions. The whole questionnaire is composed of six subscales, including spiritual growth, interpersonal relations, nutrition, physical activity, health responsibility, and stress management [32]. The questions are in a 4-point Likert scale, in which 1 to 4 indicate how often the participants had such behaviors. The alpha coefficient of the total instrument from a local study was equal to 0.91 and the range of coefficients for subscales were from 0.72 to 0.83 [33].

#### 2.4.3. Pittsburg Sleep Quality Index (PSQI)

The Pittsburgh Sleep Quality Index (PSQI) is an effective instrument used to measure the quality and patterns of sleep in different age groups. Numerous studies using the PSQI in a variety of adult populations throughout the world have supported its high validity and reliability [33]. Participants subjectively reported their sleep quality according to sleep latency, sleep duration, habitual sleep efficiency, sleep disturbances, and daytime dysfunction over the past 30 days. Questions were in Likert scale from 0 to 3, indicating the frequency and severity of the above symptoms. The English version of the PSQI was adopted among local university students in a cross-sectional study [34] and the alpha coefficient was 0.66.

#### 2.4.4. Demographic Data

Demographic characteristics such as age, gender, educational level, family household income, and program and year of study were included in this part.

### 2.5. Data Analysis

IBM SPSS version 30.0 was used for data analysis. Descriptive tables with percentages, mean scores, and standard deviations were used to summarize the demographic data of participants as well as scores from SSEIS, HPLPII, and PSQI in order to have their baseline status of EI and healthy lifestyle patterns. A structured multiphase regression analysis was used to analyze the association of demographic factors, HPLPII, and PSQI with EI in order to avoid over-adjustment of factors. After determining the hypothesized sequential association, there were 3 clusters of factors constructed. Cluster 1 variables were hypothesized to affect cluster 2 and 3 variables but not vice versa. Similarly, cluster 2 variables were assumed to affect those in cluster 3 but not vice versa. Cluster 1 included variables such as age, gender, family income, number of siblings, number of medical diagnoses, year of studying in nursing, and clinical experience. Cluster 2 related to health behaviors including cigarette and alcohol consumption, as well as all subscales in HPLPII. PSQI score > 5 and body mass index (BMI) were categorized under cluster 3. After constructing 3 clusters of variables, 3 phases of structured multiphase regression were conducted. In phase 1, a stepwise regression was undergone on cluster 1 variables only. Those significant variables in phase 1 then underwent phase 2 stepwise regression together with cluster 2. The last phase of stepwise regression was undergone on cluster 3 variables, adjusting for variables significant in both phase 1 and 2. Compared with a single-step multiple regression, this approach makes the adjustment structure explicit; compared with conventional hierarchical regression based purely on statistical block entry, the cluster ordering was guided by domain logic (background → behaviors → physiological indicators).

Regression diagnostics were examined to support model interpretation. Multicollinearity was assessed using tolerance values (reported for phases 1–3). Residual diagnostics (e.g., distribution and scatter of residuals) were inspected to check major violations of linear regression assumptions. All significance tests were two-tailed with α = 0.05, and 95% confidence intervals were reported for estimates.

## 3. Results

### 3.1. Descriptive Statistics of Demographic Characteristics, EI Level and Health Promoting Lifestyles

After 3 months of data collection, 307 eligible subjects were identified and 287 subjects completed the questionnaires at the end. The mean age among the subjects was 21.89 years (SD = 2.11, range: 18–31), with 16 (5.6%) male and 271 (94.4%) female (see Table 1). The mean SSEIS score was equal to 119.60 (95% CI = 118.57 to 120.65). Among the six dimensions in HPLPII, interpersonal relations scored the highest among other health-promoting lifestyles in the scale with mean = 2.75 (95% CI = 2.70 to 2.81). Spiritual growth and nutrition scored the second and third with mean = 2.66 (95% CI = 2.60 to 2.71) and 2.43 (95% CI = 2.38 to 2.48), respectively. The range of total score in HPLPII was 73–185. For the PSQI, the mean score was equal to 7.37 (95% CI = 7.03, 7.72) (see Table 2).

### 3.2. EI Associated Factors

As seen from the regression results, the tolerance range was 0.39 to 1.00 from phase 1 to 3 in the structured multiphase regression. In phase 1, year of studying in nursing showed significant association to EI scores among subjects (*B* = −0.96, 95% CI = −1.77 to −0.10, *p* = 0.028). It was therefore used to adjust smoking behavior, alcohol consumption, and HPLPII subscales in phase 2. Among all domains of lifestyles, only interpersonal relations (*B* = 4.42, 95% CI = 1.44 to 7.50, *p* = 0.004) and spiritual growth (*B* = 6.59, 95% CI = 3.81 to 9.37, *p* < 0.001) showed to be significant. However, significance was no longer shown in year of studying in nursing. In the last phase, the three significant variables in phase 1 and 2 adjusted PSQI and BMI. PSQI scores > 5 showed significant effects on EI (*B* = −1.95, 95% CI = −3.88 to −0.01, *p* = 0.049). Interpersonal relations (*B* = 4.45, 95% CI = 1.44 to 7.47, *p* = 0.004) and spiritual growth (*B* = 6.42, 95% CI = 3.65 to 9.20, *p* < 0.001) still remained significant, whereas BMI and year of studying in nursing appeared to have no significant effects towards EI after such adjustment (see Table 3).

## 4. Discussion

This study examined factors associated with EI and its relationship with health behaviors among prelicensure nursing students. Interpersonal relations and spiritual growth were positively associated with EI, while poor sleep quality (PSQI > 5) was negatively associated with EI. Although EI has been widely studied, our findings add to existing evidence by clarifying the modifiable health-related domains remain salient when multiple lifestyle domains and sleep quality are considered together.

Among the associated factors, interpersonal relations were found to be significant in shaping EI among nursing students. Mayer and Salovey’s ability model conceptualizes EI as the capacity to perceive, use, understand, and manage emotions [1]. Stronger interpersonal relations may reflect more frequent social interactions and support, providing opportunities to perceive and understand emotions in others and to practice emotion management during communication and conflict resolution. Effective interpersonal skills allow students to engage in meaningful discussions, seek support from peers and mentors, and resolve conflicts rationally. These abilities facilitate emotional regulation and enhance problem-solving skills, both of which are essential for professional and personal development in healthcare settings. Previous research has consistently demonstrated a strong correlation between social support and EI, as both encompass similar components, such as recognizing emotions and appraising one’s own needs as well as those of others [19]. The relationship between perceived EI and social support has been further investigated, particularly among nursing students [35]. In a study exploring help-seeking behaviors, students who sought guidance from family or peers when faced with academic or professional challenges were more likely to develop successful social networks. This in turn contributed to higher levels of emotional understanding, reinforcing the notion that strong interpersonal connections positively impact EI [14,35]. Furthermore, social networking has been shown to play a crucial role in stress management among nursing students. Effective social support can mitigate psychological distress and reduce stress-induced effects. Findings from previous studies indicate that nursing students with greater social support experience significantly fewer stress-related psychological symptoms, demonstrating the protective nature of well-developed interpersonal relationships in emotionally demanding environments [14,36]. Individuals with satisfying interpersonal relationships are more likely to experience emotional well-being and greater resilience to stress. As EI enables individuals to understand and regulate emotions effectively, this in turn facilitates productive thinking and rational decision-making in social settings [37]. The reciprocal nature of this relationship suggests that fostering strong social support networks leads to enhanced EI, while higher EI further strengthens interpersonal connections [35]. Nursing students who possess the ability to manage social challenges effectively are more likely to develop positive interactions, contributing to an improved overall social environment [38].

Spiritual growth was identified as another significant factor influencing EI among nursing students. It encompasses an individual’s ability to explore life’s purpose, establish long-term goals, and cultivate open-mindedness toward challenges [33]. EI, which includes the ability to understand and regulate emotions, strongly relies on traits such as personal growth, self-discipline, and self-mastery [39]. Spiritual growth involving meaning-making and purpose-oriented development may align with the ‘management’ component of EI in Mayer and Salovey’s ability model [1] by supporting reflective coping and regulation in emotionally demanding learning environments. Spiritual development enhances EI by promoting emotional self-regulation and fostering inner peace, allowing individuals to respond to challenges with composure. This regulation of emotions is particularly beneficial for nursing students, who must navigate demanding clinical environments while managing patient care effectively. Additionally, spiritual growth nurtures empathy and compassion, strengthening one’s ability to connect with others on a deeper level. By cultivating spiritual awareness, nursing students can improve their emotional well-being, develop resilience, and engage in social interactions with greater sensitivity and understanding. In healthcare settings, where nurses frequently encounter emotionally challenging situations, spirituality serves as a valuable coping mechanism. It empowers individuals to find meaning in their work, maintain emotional balance, and engage in compassionate caregiving. Furthermore, the positive influence of spiritual development on EI reinforces the importance of incorporating relevant training into nursing education to enhance students’ ability to process emotions effectively.

Although no studies specifically examine the relationship between EI and sleep deprivation in nursing education, relevant research exploring sleep’s impact on emotional intelligence in healthy adults provides valuable insights. A study investigating the effects of sleep deprivation revealed that individuals who lacked sufficient rest exhibited significantly lower EI scores, particularly in domains related to social functioning and stress management [23]. From a physiological perspective, sleep deprivation negatively affects the brain’s ability to process emotions. The amygdala, which is responsible for emotional regulation, becomes hyperactive in sleep-deprived individuals when exposed to negative visual stimuli. In contrast, those who receive adequate rest exhibit normal amygdala activity [40]. This suggests that insufficient sleep disrupts emotional regulation, leading to heightened emotional reactivity and impaired decision-making. For nursing students, maintaining a healthy sleep schedule is essential for emotional well-being and professional effectiveness. Given the emotionally demanding nature of nursing, inadequate sleep may compromise students’ ability to manage stress, engage in meaningful interpersonal interactions, and maintain a balanced emotional state. Addressing sleep hygiene through structured educational programs can help nursing students improve their EI and overall psychological resilience.

This study also examined demographic characteristics—including age, gender, and educational background—but found no significant differences in EI levels among nursing students. These findings contradict previous studies that suggest demographic variables may influence EI [5,12,41]. The discrepancy may be attributed to the relatively small sample size and narrow age range of participants in this study, which could limit the generalizability of the findings. Importantly, the sample was predominantly female, which limits statistical power to examine gender differences and restricts generalizability to more gender-balanced cohorts. Despite these inconsistencies, existing literature suggests that factors such as professional experience and cultural influences may shape EI. Norms around emotional expression, interpersonal harmony, and coping practices can vary across cultural settings, potentially influencing EI development. Future research should investigate broader demographic variables to determine whether EI levels differ based on socioeconomic background, clinical exposure, or personal experiences.

The results explained approximately one-third of the variance in EI (R^2^~32%), indicating substantial unexplained variance. Other determinants not captured here, such as personality traits, stress, coping style, quality of social support, resilience, or learning environment factors, may also influence EI and warrant inclusion in future research.

For limitations, data collection was only restricted to one local nursing school. Although the collection site chosen was one of the biggest nursing schools with the longest history in Hong Kong, the generalizability of this study was still limited. Other nursing education institutes can be recruited to expand the study results in future studies. Nevertheless, all primary measures were self-reported, raising the possibility of common method bias and social desirability effects, particularly among health students. Although anonymity and sealed-envelope returns were used to reduce response pressure, method variance cannot be ruled out. Future studies should consider multi-source data (e.g., objective sleep indicators, behavioral measures, or peer/educator ratings) and longitudinal/intervention designs to better establish directionality. In addition, PSQI internal consistency reported in a local study (α = 0.66) suggests measurement error that may attenuate associations involving sleep quality. Future studies should use multi-site sampling, longitudinal or interventional designs, and objective or multi-source measures to strengthen inference.

## 5. Conclusions

Enhancement of EI in nursing education is important, and this study identified interpersonal relations, spiritual growth, and sleep quality as factors associated with EI among prelicensure nursing students. These findings may inform student support strategies and EI-related educational initiatives that integrate interpersonal skill-building, reflective/purpose-oriented growth, and sleep hygiene awareness. Future longitudinal and intervention research is needed to clarify causality and evaluate whether targeting these domains improves EI over time.

## Figures and Tables

**Table 1:** Participants’ demographic characteristics.

	Total (N = 287)
**Age (mean+/−SD)**	21.89 ± 2.11 (18–31)
**Gender** Male Female	16 (5.6%) 271 (94.4%)
**Mode of study** Full-time Part-time	172 (59.9%) 115 (40.1%)
**Height (cm) (mean+/−SD)**	160.99 ± 6.08 (150–180)
**Weight (kg) (mean+/−SD)**	52.52 ± 8.22 (40–95)
**Body mass index (kg/m^2^) (mean+/−SD)**	20.22 ± 2.49 (15.60–32.40)
**Cigarette consumption:**	
Ever-smokers	11 (3.8%)
Non-smokers	276 (96.2%)
**Alcohol consumption:**	
Drinkers	129 (44.9%)
Non-drinkers	158 (55.1%)
**Educational level before entry of nursing school:**	
Secondary school or below	96 (33.4%)
Higher diploma	95 (33.2%)
Associated degree	50 (17.4%)
Bachelor degree or above	46 (16.0%)
**Marital Status:**	
Single	284 (99%)
Married	3 (1%)
**Monthly Family household income:**	
<$10,000	12 (4.2%)
$10,000–$30,000	115 (40.1%)
$30,000–$60,000	110 (38.3%)
$60,000–$100,000	40 (13.9%)
>$100,000	10 (3.5%)
**Number of siblings:**	
0	65 (22.6%)
1	135 (47.0%)
2	55 (19.2%)
3	16 (5.6%)
>3	16 (5.6%)
**Ranking among siblings:**	
Top	91 (31.7%)
Second	95 (33.1%)
Third	30 (10.5%)
Forth or above	6 (2.1%)
Only child	65 (22.6%)
**Personal medical history**	
Nil	252 (87.8%)
More than 1	35 (12.2%)
**Year of study in Nursing:**	
1	97 (33.8%)
2	75 (26.1%)
3	62 (21.6%)
4	33 (11.5%)
5	20 (7.0%)
**Do you have any clinical experience?**	
Yes	274 (95.5%)
No	13 (4.5%)
**Cumulative grade point average (GPA) (*n* = 158)**	3.05 ± 0.43 (2.00–4.00)

**Table 2:** Participants’ health-promoting lifestyle.

Variables		Mean ± SD
HPLP II		
Health responsibility		2.14 ± 0.49
Physical activity		2.04 ± 0.55
Nutrition		2.43 ± 0.42
Spiritual growth		2.66 ± 0.05
Interpersonal relations		2.75 ± 0.45
Stress management		2.39 ± 0.43
Total score		125.29 ± 19.36
**PSQI score**		7.37 ± 2.97

**Table 3:** Associated factors of EI among nursing students (N = 287).

Variables	Estimates	95% CI		*p*
**^a^ Phase 1 (R^2^ = 1.7%, adjusted R^2^ = 1.3%)**				
Year of studying in nursing	−0.96	−1.77, −0.10		0.028 *
**^b^ Phase 2 (R^2^ = 31.8%, adjusted R^2^ = 31.1%) (adjusted by year of studying in nursing)**				
HPLPII domains:				
⮚ Spiritual growth	6.59	3.81, 9.37		<0.001 **
⮚ Interpersonal relations	4.42	1.44, 7.50		0.004 **
**^c^ Phase 3 (R^2^ = 32.8%, adjusted R^2^ = 31.8%) (adjusted by year of studying in nursing, spiritual growth and interpersonal relations)**				
PSQI score > 5	−1.95	−3.88, −0.01		0.049 *

^a^ Phase 1 excluded variables: age, gender, educational level before entry of nursing school, marital status, monthly family household income, number of siblings, ranking among siblings, number of medical diagnoses, and clinical experience. ^b^ Phase 2 excluded variables: cigarette consumption, alcohol consumption, and HPLP subscales (health responsibility, physical activity, nutrition, and stress management). ^c^ Phase 3 excluded variables: BMI. * *p* < 0.005, ** *p* < 0.001.

## Data Availability

All data are contained within the article.

## Public Involvement Statement

No public involvement in any aspect of this research.

## Guidelines and Standards Statement

This manuscript was drafted against the STROBE reporting guideline for cross-sectional research.
